# Supplementary material for: X-intNMF: a cross- and intra-omics regularized NMF framework for multi-omics integration
Source: Bioinformatics. 2026 Jan 27;42(2):btag046. doi: 10.1093/bioinformatics/btag046 (PMC12910379; doi:10.1093/bioinformatics/btag046)
Supplement: btag046_Supplementary_Data [file btag046_supplementary_data.pdf]

Support Material

Tien-Thanh Bui, Rui Xie, and Wei Zhang

January 15, 2026

1 Notation Guideline

- All variables in **bold**, including those with a single index, represent either vectors or matrices. The bold numerals **0** and **1** denote zero and one vectors or matrices, depending on the context.
- All variables in non-bold form, including those with two indices (for matrices) or a single index (for vectors), represent scalar values.
- Superscripts enclosed in parentheses indicate indexing within a list or block of matrices. Specifically, a single integer in the superscript (e.g., $\mathbf{X}^{(d)}$) refers to the dth matrix in a list. A superscript with two integers (e.g., $\mathbf{X}^{(dp)}$) denotes the element at the dth row and p^{th} column in a matrix block.

All mathematical notations used in the paper are summarized in Table S1.

Name	Type	Definition
D, N	Input	Number of omics layers, Number of samples
m_d	Input	Feature size of dth omic layer
M	Inferred	Total number of features
$\mathbf{X}^{(d)}$	Input	$(N \times m_d)$-shaped matrix of dth omic layer
$\overline{\mathbf{X}}$	Inferred	$(N \times M)$-shaped concatenation of all omics layers along the feature axis.
$\mathbf{A}$	Input	$(M \times M)$-shaped adjacency matrix of the cross-intraomics feature interaction network
$\tilde{\mathbf{A}}$	Inferred	$(M \times M)$-shaped normalized adjacency matrix of $\mathbf{A}$
$\mathbf{\Delta}, \mathbf{\Delta}^{-1/2}$	Inferred	$(M \times M)$-shaped degree matrix of $\mathbf{A}$ and its normalized form
$\mathbf{L}$	Inferred	$(M \times M)$-shaped Laplacian matrix of the interaction network $\mathbf{A}$
$\mathbf{A}^{(pq)}$	Inferred	$(m_p \times m_q)$-shaped interaction network adjacency matrix between p^{th} and q^{th} omic layers
$\mathbf{L}^{(pq)}$	Inferred	$(m_p \times m_q)$-shaped partition matrix of $\mathbf{L}$
α	Hyperparameter	Interaction regularization parameter
β_d	Hyperparameter	Omic-specific sparsity regularization parameters
γ_i	Hyperparameter	Sample-specific sparsity regularization parameters
K	Hyperparameter	Number of latent components
$\mathbf{W}^{(d)}$	Output	$(m_d \times K)$-shaped d^{th} omic factor matrix
$\mathbf{H}$	Output	$(K \times N)$-shaped sample factor matrix
$\boldsymbol{\lambda}$	Inferred	Regression coefficients in Cox proportional hazards model
ξ, η	Hyperparameter	Shrinkage control and l_1/l_2-norm mixing parameters in Cox proportional hazards model

Table S1: List of mathematical notations used in the paper.

2 Optimization of the X-IntNMF model

Since the objective function is not joinly convex over $\mathbf{W}^{(d)}$ and $\mathbf{H}$, it must be solved iteratively by optimizing one matrix while keeping the other fixed. Recall the the optimization problem of X-IntNMF:

$$\begin{aligned} \min_{\mathbf{H},\mathbf{W}^{(1)},\mathbf{W}^{(2)},...,\mathbf{W}^{(D)}} & f(\mathbf{H},\mathbf{W}^{(1)},\mathbf{W}^{(2)},...,\mathbf{W}^{(D)}) \\ \text{s.t. } & \mathbf{W}^{(d)} \in \mathbb{R}_+^{m_d\times K} \text{ for } d \in \{1,2,...,D\} \\ & \mathbf{H} \in \mathbb{R}_+^{K\times N}. \end{aligned} \tag{2.1}$$

where

$$\begin{aligned} f(\mathbf{H},\mathbf{W}^{(1)},\mathbf{W}^{(2)},...,\mathbf{W}^{(D)}) = & \frac{1}{2}\sum_{d=1}^{D}\left\|\mathbf{X}^{(d)} - \mathbf{W}^{(d)}\mathbf{H}\right\|_F^2 \\ & + \frac{\alpha}{2}\sum_{p=1}^{D}\sum_{q=1}^{D}\text{Tr}(\mathbf{W}^{(p)T}\mathbf{L}^{(pq)}\mathbf{W}^{(q)}) \\ & + \sum_{d=1}^{D}\beta_d\left\|\mathbf{W}^{(d)}\right\|_1 + \sum_{i=1}^{N}\gamma_i\left\|\mathbf{H}_{\cdot,i}\right\|_1 \end{aligned} \tag{2.2}$$

Let $\mathbf{\Phi}^{(d)} = [\phi_{ij}]^{(d)}$ and $\mathbf{\Psi} = [\psi_{ij}]$, the Lagrange multipliers $\mathcal{L}$ for f is defined as (2.3):

$$\mathcal{L} = f + \text{Tr}(\mathbf{\Psi}\mathbf{H}^T) + \sum_{d=1}^{D}\text{Tr}(\mathbf{\Phi}^{(d)}\mathbf{W}^{(d)T}) \tag{2.3}$$

Equation (2.4) and (2.5) represent the partial derivatives of $\mathcal{L}$ with respect to $\mathbf{H} \in \mathbb{R}_+^{K\times N}$ and $\mathbf{W}^{(d)} \in \mathbb{R}_+^{m_d\times K}$:

$$\frac{\partial\mathcal{L}}{\partial\mathbf{W}^{(d)}} = (\mathbf{W}^{(d)}\mathbf{H} - \mathbf{X}^{(d)})\mathbf{H}^T + \alpha\sum_{p=1}^{D}\mathbf{L}^{(dp)}\mathbf{W}^{(p)} + \beta_d\mathbf{1} + \mathbf{\Phi}^{(d)} \tag{2.4}$$

$$\frac{\partial\mathcal{L}}{\partial\mathbf{H}} = \sum_{d=1}^{D}\mathbf{W}^{(d)T}(\mathbf{W}^{(d)}\mathbf{H} - \mathbf{X}^{(d)}) + \mathbf{\Psi} + \mathbf{1}\boldsymbol{\gamma} \tag{2.5}$$

Using Karush-Kuhn-Tucker (KKT) conditions $W_{ij}^{(d)}\phi_{ij}^{(d)} = 0$, $H_{ij}\psi_{ij} = 0$, $\frac{\partial\mathcal{L}}{\partial\mathbf{W}^{(d)}} = \mathbf{0}$, and $\frac{\partial\mathcal{L}}{\partial\mathbf{H}} = \mathbf{0}$, the update rules for $\mathbf{W}^{(d)}$ and $\mathbf{H}$ can be derived in Section 2.1 and 2.2 respectively.

2.1 For omics factor matrices

From Equation (2.4) and condition of $\frac{\partial\mathcal{L}}{\partial\mathbf{W}^{(d)}} = \mathbf{0}$, the following equation can be derived:

$$(\mathbf{W}^{(d)}\mathbf{H} - \mathbf{X}^{(d)})\mathbf{H}^T + \alpha\sum_{p=1}^{D}\mathbf{L}^{(dp)}\mathbf{W}^{(p)} + \beta_d\mathbf{1} + \mathbf{\Phi}^{(d)} = \mathbf{0} \tag{2.6}$$

Swap $\mathbf{\Phi}^{(d)}$ to the right side and reverse the sign of Equation (2.6), the following equation can be derived:

$$\mathbf{\Phi}^{(d)} = (\mathbf{X}^{(d)} - \mathbf{W}^{(d)}\mathbf{H})\mathbf{H}^T - \alpha \sum_{p=1}^{D} \mathbf{L}^{(dp)}\mathbf{W}^{(p)} - \beta_d \mathbf{1} \tag{2.7}$$

Since $\mathbf{L}^{(dp)} = \mathbf{I}^{(dp)} - \tilde{\mathbf{A}}^{(dp)}$, Equation (2.7) can be rewritten as:

$$\begin{aligned}
\mathbf{\Phi}^{(d)} &= (\mathbf{X}^{(d)} - \mathbf{W}^{(d)}\mathbf{H})\mathbf{H}^T - \alpha \sum_{p=1}^{D} (\mathbf{I}^{(dp)} - \tilde{\mathbf{A}}^{(dp)})\mathbf{W}^{(p)} - \beta_d \mathbf{1} \\
&= (\mathbf{X}^{(d)} - \mathbf{W}^{(d)}\mathbf{H})\mathbf{H}^T + \alpha \sum_{p=1}^{D} (\tilde{\mathbf{A}}^{(dp)} - \mathbf{I}^{(dp)})\mathbf{W}^{(p)} - \beta_d \mathbf{1} \\
&= \left(\mathbf{X}^{(d)}\mathbf{H}^T + \alpha \sum_{p=1}^{D} \tilde{\mathbf{A}}^{(dp)}\mathbf{W}^{(p)} \right) - \left(\mathbf{W}^{(d)}\mathbf{H}\mathbf{H}^T + \alpha \sum_{p=1}^{D} \mathbf{I}^{(dp)}\mathbf{W}^{(p)} + \beta_d \mathbf{1} \right)
\end{aligned} \tag{2.8}$$

where $\mathbf{I}^{(dp)}$ is the block at row d and column p of the identity matrix. However, $\mathbf{I}^{(dp)}$ only non-zero when $d = p$, then:

$$\begin{aligned}
\mathbf{\Phi}^{(d)} &= \left(\mathbf{X}^{(d)}\mathbf{H}^T + \alpha \sum_{p=1}^{D} \tilde{\mathbf{A}}^{(dp)}\mathbf{W}^{(p)} \right) - \left(\mathbf{W}^{(d)}\mathbf{H}\mathbf{H}^T + \alpha \mathbf{W}^{(d)} + \beta_d \mathbf{1} \right) \\
&\equiv \mathbf{U}^{(d)} - \mathbf{V}^{(d)}
\end{aligned} \tag{2.9}$$

Combining Equation (2.9) with the KKT condition $W_{ij}^{(d)} \phi_{ij}^{(d)} = 0$, the following equation can be derived:

$$\Phi_{ij}^{(d)} W_{ij}^{(d)} = (U_{ij}^{(d)} - V_{ij}^{(d)}) W_{ij}^{(d)} = 0 \tag{2.10}$$

The update rule for $\mathbf{W}^{(d)}$ can be derived from Equation (2.10) as follows:

$$W_{ij}^{(d)} \leftarrow W_{ij}^{(d)} \times \frac{U_{ij}^{(d)}}{V_{ij}^{(d)}} = W_{ij}^{(d)} \times \frac{\left[\mathbf{X}^{(d)}\mathbf{H}^T + \alpha \sum_{p=1}^{D} \tilde{\mathbf{A}}^{(dp)}\mathbf{W}^{(p)} \right]_{ij}}{\left[\mathbf{W}^{(d)}\mathbf{H}\mathbf{H}^T + \alpha \mathbf{W}^{(d)} \right]_{ij} + \beta_d} \tag{2.11}$$

2.2 For sample factor matrices

From Equation (2.5) and condition of $\frac{\partial \mathcal{L}}{\partial \mathbf{H}} = \mathbf{0}$, the following equation can be derived:

$$\sum_{d=1}^{D} \mathbf{W}^{(d)T}(\mathbf{W}^{(d)}\mathbf{H} - \mathbf{X}^{(d)}) + \mathbf{\Psi} + \mathbf{1}\boldsymbol{\gamma} = \mathbf{0} \tag{2.12}$$

Swap $\mathbf{\Psi}$ to the right side and reverse the sign of Equation (2.12), the following equation can be derived:

$$\begin{aligned}
\mathbf{\Psi} &= \sum_{d=1}^{D} \mathbf{W}^{(d)T}(\mathbf{X}^{(d)} - \mathbf{W}^{(d)}\mathbf{H}) - \mathbf{1}\boldsymbol{\gamma} \\
&= \left(\sum_{d=1}^{D} \mathbf{W}^{(d)T}\mathbf{X}^{(d)} \right) - \left(\sum_{d=1}^{D} \mathbf{W}^{(d)T}\mathbf{W}^{(d)}\mathbf{H} + \mathbf{1}\boldsymbol{\gamma} \right) \\
&\equiv \mathbf{S} - \mathbf{T}
\end{aligned} \tag{2.13}$$

Combining Equation (2.13) with the KKT condition $H_{ij}\psi_{ij} = 0$, the following equation can be derived:

$$\Psi_{ij}H_{ij} = (S_{ij} - T_{ij})H_{ij} = 0 \tag{2.14}$$

The update rule for $\mathbf{H}$ can be derived from Equation (2.14) as follows:

$$H_{ij} \leftarrow H_{ij} \times \frac{S_{ij}}{T_{ij}} = H_{ij} \times \frac{\left[\sum_{d=1}^{D} \mathbf{W}^{(d)T}\mathbf{X}^{(d)}\right]_{ij}}{\left[\sum_{d=1}^{D} \mathbf{W}^{(d)T}\mathbf{W}^{(d)}\mathbf{H}\right]_{ij} + \gamma_i} \tag{2.15}$$

2.3 Algorithm

The algorithm for the X-IntNMF model is summarized in Algorithm S1.

Algorithm S1: X-IntNMF algorithm

Input: $\mathbf{X}^{(d)}$, α, β_d, γ_i, K
Output: $\mathbf{W}^{(d)}$, $\mathbf{H}$

Normalize $\mathbf{X}^{(d)}$ to $[0, 1]$;
Initialize $\mathbf{W}^{(d)}$ and $\mathbf{H}$;
while *not converged* **do**
  for $d = 1$ **to** D **do**
    for $i = 1$ **to** m_d **do**
      for $j = 1$ **to** K **do**
        $W_{ij}^{(d)} \leftarrow W_{ij}^{(d)} \times \frac{\left[\mathbf{X}^{(d)}\mathbf{H}^T + \alpha \sum_{p=1}^{D} \tilde{\mathbf{A}}^{(dp)}\mathbf{W}^{(p)}\right]_{ij}}{\left[\mathbf{W}^{(d)}\mathbf{H}\mathbf{H}^T + \alpha\mathbf{W}^{(d)}\right]_{ij} + \beta_d}$;
      end
    end
  end
  for $i = 1$ **to** N **do**
    for $j = 1$ **to** K **do**
      $H_{ij} \leftarrow H_{ij} \times \frac{\left[\sum_{d=1}^{D} \mathbf{W}^{(d)T}\mathbf{X}^{(d)}\right]_{ij}}{\left[\sum_{d=1}^{D} \mathbf{W}^{(d)T}\mathbf{W}^{(d)}\mathbf{H}\right]_{ij} + \gamma_i}$;
    end
  end
end
return $\mathbf{W}^{(1)}, \mathbf{W}^{(2)}, ..., \mathbf{W}^{(D)}, \mathbf{H}$;

3 3-omics classification experimental results

Methods	ER		PR		HER2		TN	
	MCC	AUC	MCC	AUC	MCC	AUC	MCC	AUC
X-intMF	0.7283	**0.9486**	0.6980	0.8840	**0.2569**	**0.7472**	**0.6606**	0.9475
X-intMF ($\alpha = 0$)	0.7283	**0.9486**	0.6980	0.8840	0.2075	0.6897	**0.6606**	0.9475
MOGONET	0.5300	0.8674	0.5445	0.8433	-0.0049	0.5929	0.4173	0.8373
MCRGCN	-0.0118	0.4861	-0.0130	0.4858	0.0026	0.5390	-0.0071	0.4980
MOFA2	0.6995	0.9302	0.6908	0.8699	0.1355	0.7108	0.6215	0.9337
iNMF	0.6475	0.9155	0.6021	0.8407	0.0778	0.5866	0.5967	0.9298
iGMFNA	**0.7532**	0.9479	**0.7383**	**0.9006**	0.2504	0.7237	0.6482	**0.9511**

Table S2: Breast cancer phenotype classification results using the 3-omics setting. Bold values indicate the best performance for each metric.

Methods	Survival: $<18/\geq18$		Disease-free: $\leq12/\geq18$	
	MCC	AUC	MCC	AUC
X-intMF	0.1132	0.6505	**0.3100**	**0.7210**
X-intMF ($\alpha = 0$)	-0.0266	0.5134	0.1703	0.6421
MOGONET	0.0137	0.5505	-0.0130	0.4705
MCRGCN	-0.0026	0.5070	0.0053	0.5105
MOFA2	-0.0040	0.6237	0.0888	0.6993
iNMF	**0.1429**	0.6420	0.0610	0.5595
iGMFNA	0.0478	**0.6535**	0.0000	0.5855

Table S3: Ovarian cancer survival and cancer-free duration range classification results using the 3-omics setting. Bold values indicate the best performance for each task.

4 Survival analysis results for BRCA, LUAD, and OV

The following figures present the Kaplan-Meier survival analysis results of the 2-omics and 3-omics datasets. All figures follow the same format: The input data used for the Cox model in (a) is the sample factor matrix $\mathbf{H}$ from the proposed framework, (b) is the sample factor matrix $\mathbf{H}$ from the ablated model with $\alpha = 0$, and (c) is from the original input data.

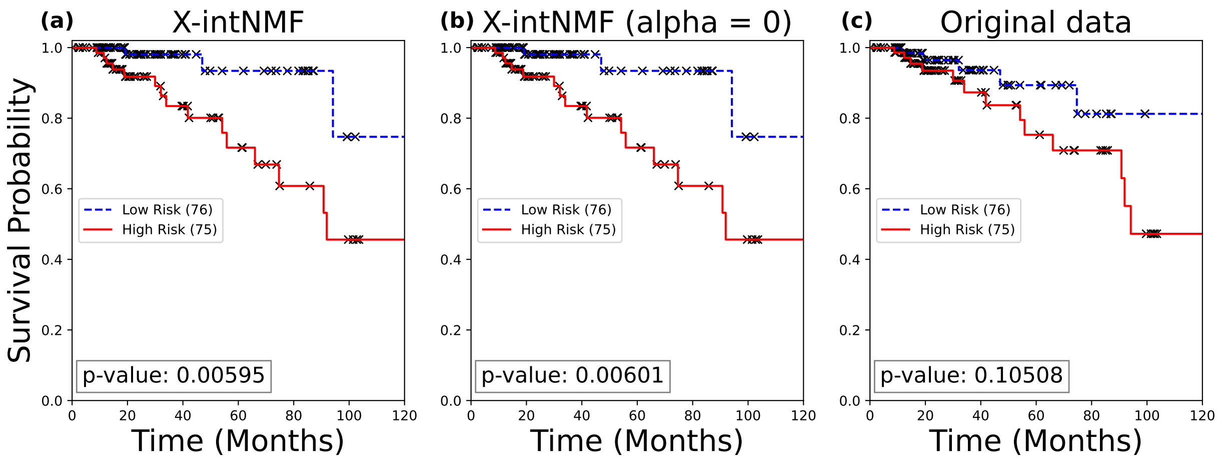

Figure S1: Kaplan-Meier survival analysis of the breast cancer dataset using the 2-omics setting.

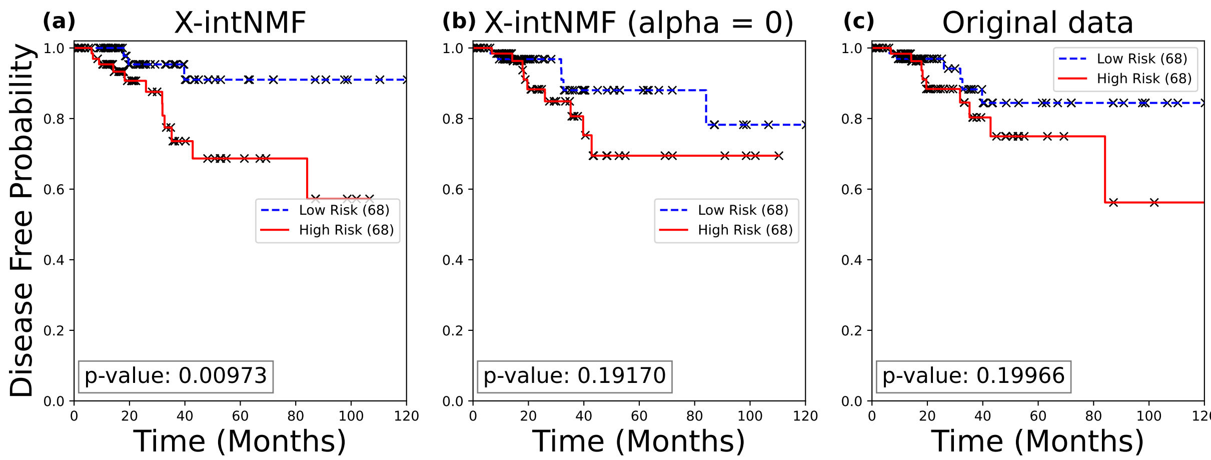

Figure S2: Kaplan-Meier disease-free analysis of the breast cancer dataset using the 2-omics setting.

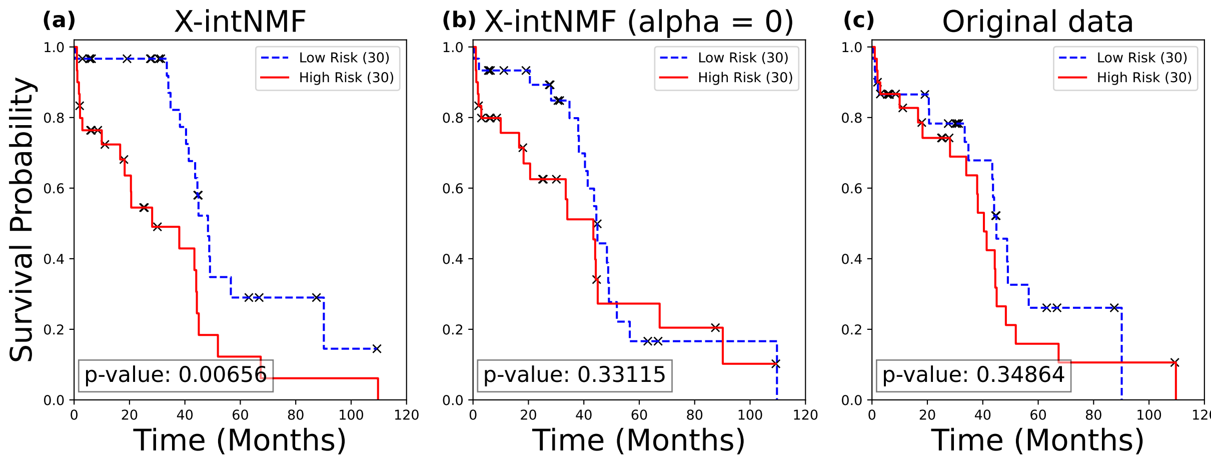

Figure S3: Kaplan-Meier survival analysis of the ovarian cancer dataset using the 2-omics setting.

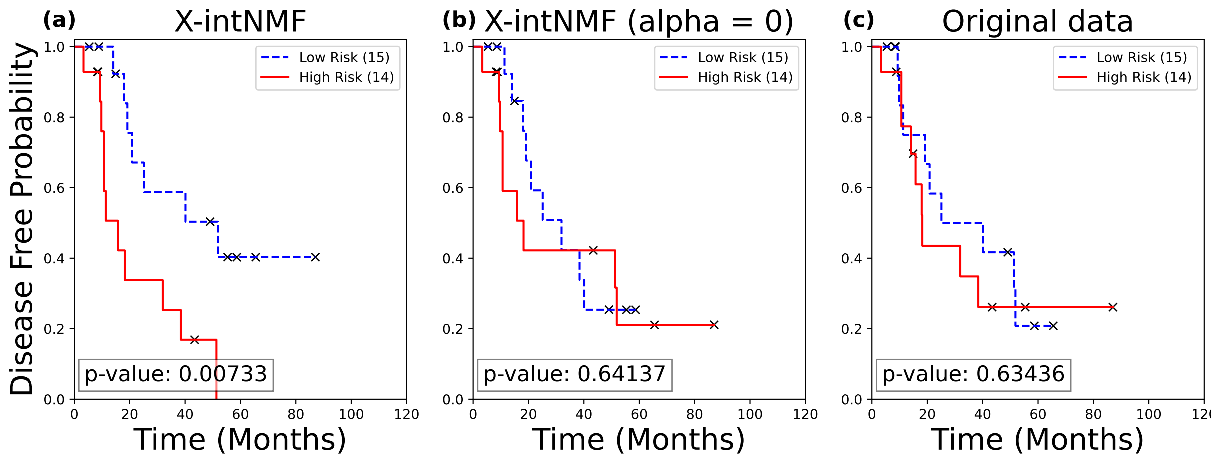

Figure S4: Kaplan-Meier disease-free analysis of the ovarian cancer dataset using the 2-omics setting.

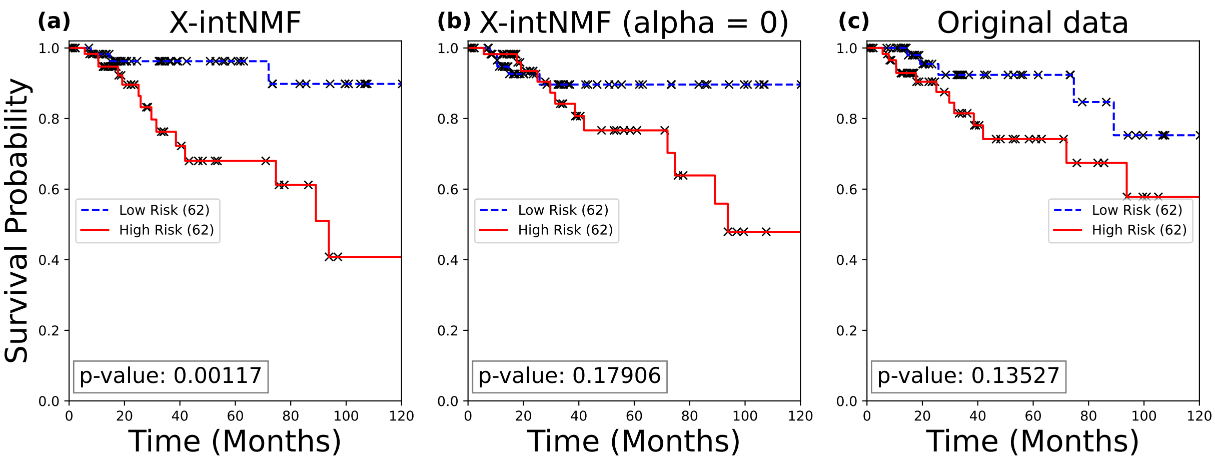

Figure S5: Kaplan-Meier survival analysis of the breast cancer dataset using the 3-omics setting.

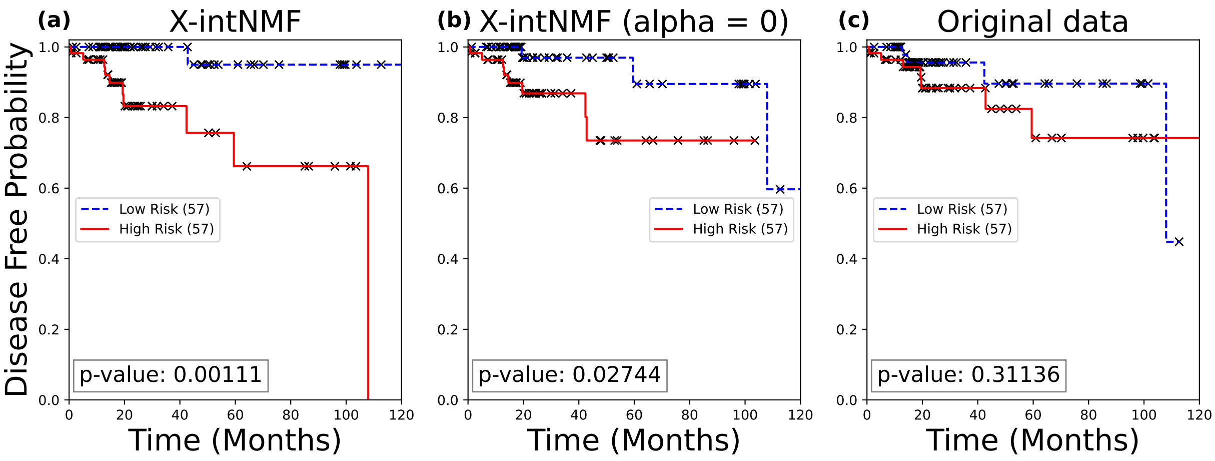

Figure S6: Kaplan-Meier disease-free analysis of the breast cancer dataset using the 3-omics setting.

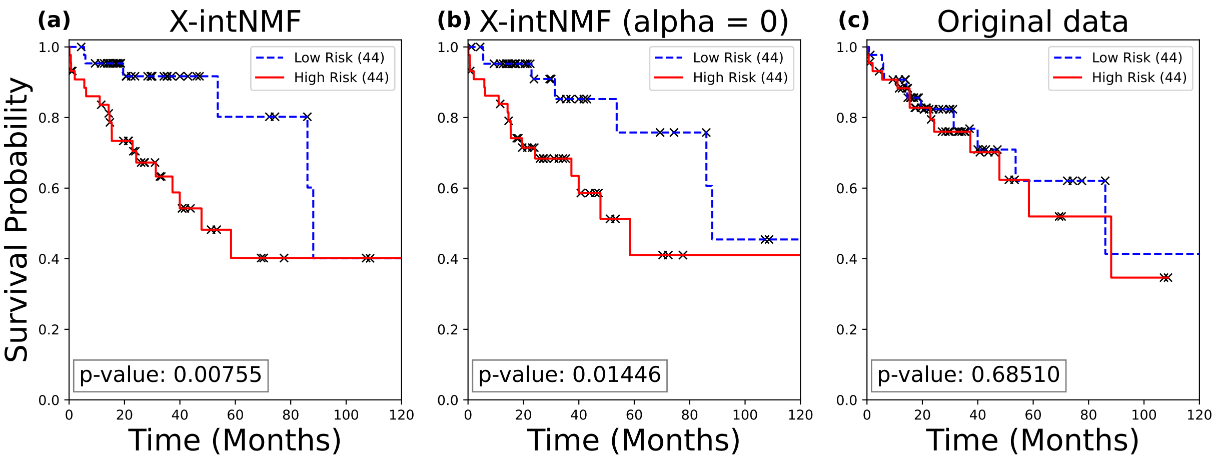

Figure S7: Kaplan-Meier survival analysis of the lung cancer dataset using the 3-omics setting.

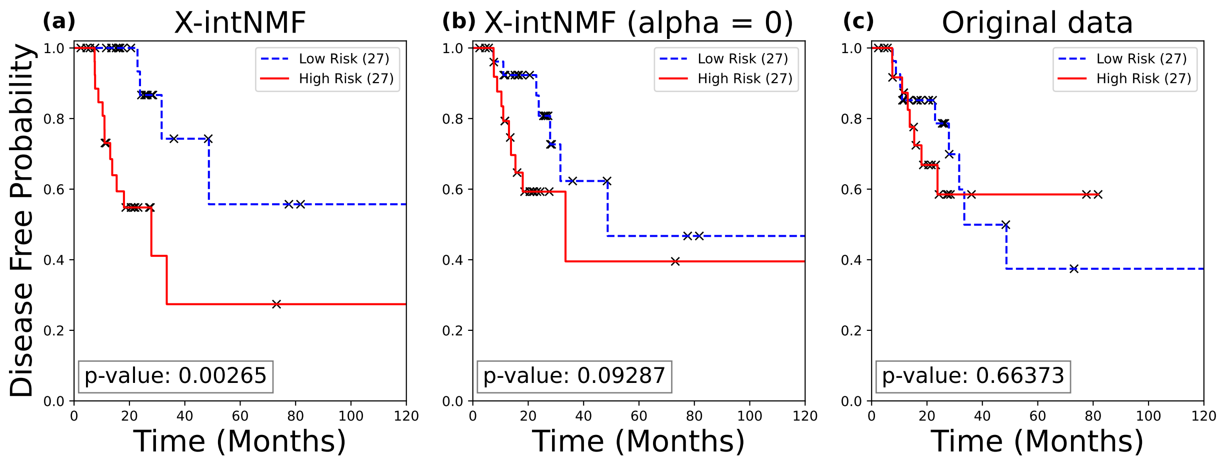

Figure S8: Kaplan-Meier disease-free analysis of the lung cancer dataset using the 3-omics setting.

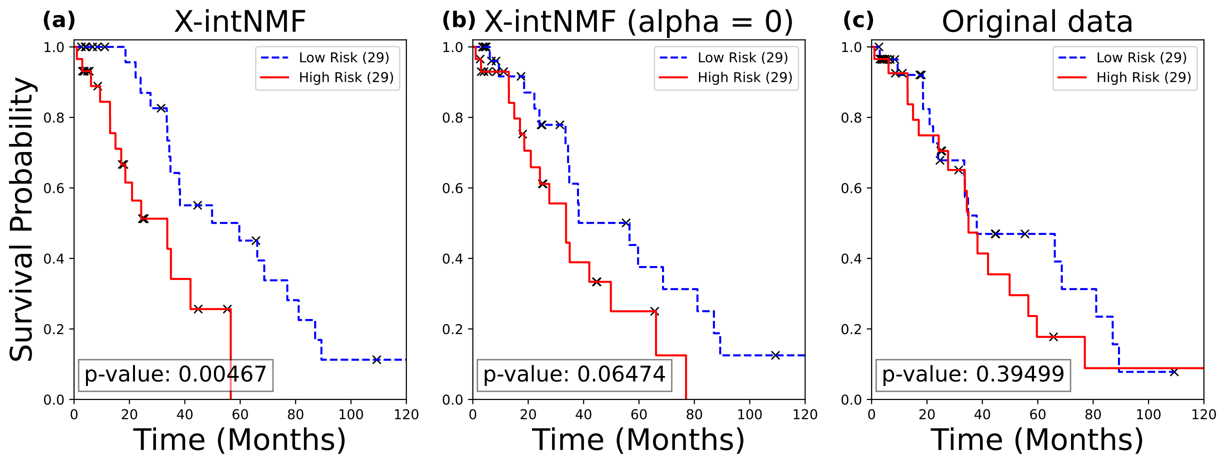

Figure S9: Kaplan-Meier survival analysis of the ovarian cancer dataset using the 3-omics setting.

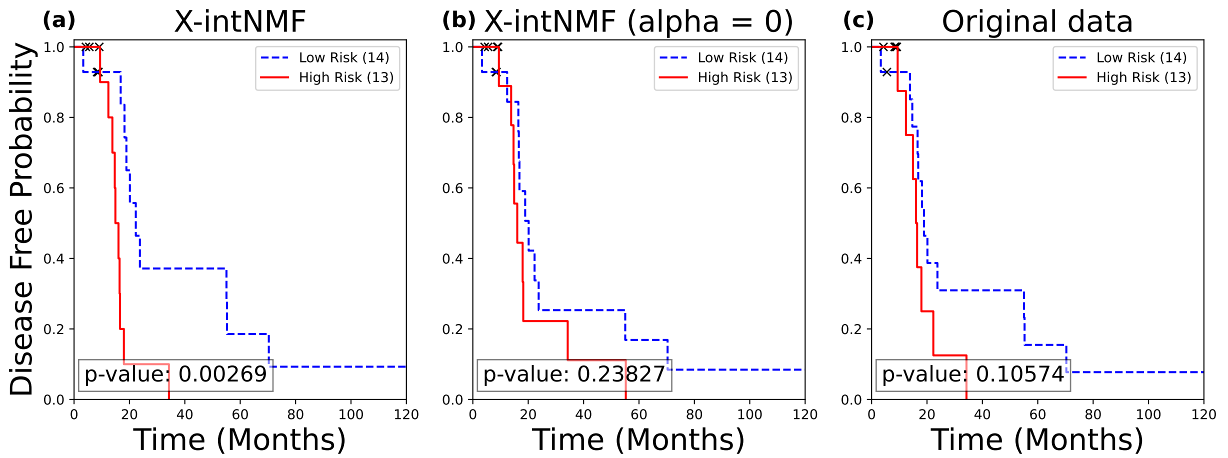

Figure S10: Kaplan-Meier disease-free analysis of the ovarian cancer dataset using the 3-omics setting.

5 Convergence logs over iterations

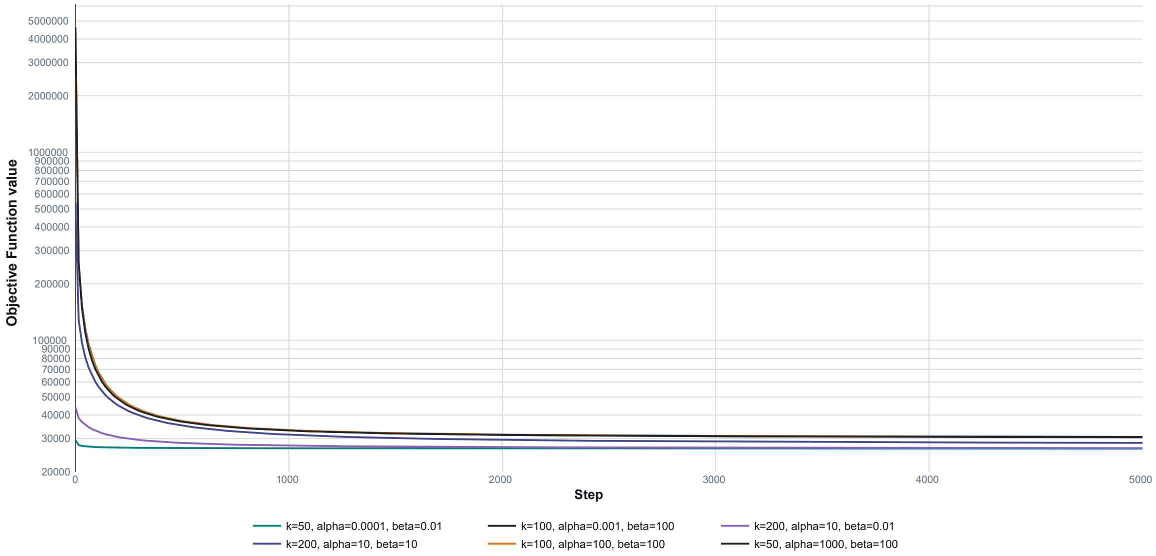

Figure S11: Convergence logs of the objective function under different parameter settings across iterations for the 2-omics breast cancer dataset, recorded using MLFlow.

6 Simulation studies

To further validate the effectiveness of the proposed X-IntNMF model, we conducted simulation studies on synthetic datasets. The goal of this experiment is to evaluate the model's ability to recover known underlying cross-omic interaction within the noisy data using graph-guided regularization. Additional mathematical notations exclusively for this experiment are illustrated in Table S4.

Name	Definition
$\overline{\mathbf{X}^{(d)}}$	Original structure of dth omic layer
$\overline{\mathbf{W}^{(d)}}$	Original structure of the $(m_d \times K)$-shaped d^{th} omic factor matrix
$\overline{\mathbf{H}}$	Original structure of the $(K \times N)$-shaped sample factor matrix
$\mathbf{X}^{(d)}$	$(N \times m_d)$-shaped matrix of dth omic layer
$\mathbf{W}^{(d)}$	$(m_d \times K)$-shaped d^{th} omic factor matrix
$\mathbf{H}$	$(K \times N)$-shaped sample factor matrix
$\widehat{\mathbf{W}}^{(d)}$	$(m_d \times K)$-shaped d^{th} omic factor matrix, predicted from $\mathbf{X}^{(d)}$
$\widehat{\mathbf{H}}$	$(K \times N)$-shaped sample factor matrix, predicted from $\mathbf{X}^{(d)}$
$\widehat{\mathbf{A}}$	Predicted binary interaction matrix between two omic layers

Table S4: List of additional mathematical notations used in the simulation studies.

To be specific, we can formulate the problem as follow: Suppose i and j are two features in two different omic layers d_1 and d_2. If they have interaction, then we want the model to capture this similarity, i.e., $\widehat{\mathbf{W}}^{(d_1)}_{i,:}$ and $\widehat{\mathbf{W}}^{(d_2)}_{j,:}$ should also be similar.

The detailed experimental setup and results are presented as follows:

1. **Step 1: Generate the binary interaction matrix.** Construct a binary matrix $\mathbf{A}^{(12)} \in \{0,1\}^{m_1 \times m_2}$, assuming without loss of generality that $m_1 \leq m_2$. Each column of $\mathbf{A}^{(12)}$ contains exactly one entry

with $A_{ij}^{(12)} = 1$ and zeros elsewhere. This ensures that each feature in the second omic uniquely interacts with a feature in the first omic, simplifying the generation of the original structures $\overline{\mathbf{W}^{(1)}}$ and $\overline{\mathbf{W}^{(2)}}$.

2. **Step 2: Generate the original feature matrices.** Based on $\mathbf{A}^{(12)}$, for each interacting pair (i, j) where $A_{ij}^{(12)} = 1$, set $\overline{\mathbf{W}}_{i,:}^{(1)}$ and $\overline{\mathbf{W}}_{j,:}^{(2)}$ to be identical random sparse binary vectors $\mathbf{w} \in \{0, 1\}^K$, ensuring a cosine similarity of 1 for interacting pairs while minimizing similarity among non-interacting pairs. We set $K = 50$ with a sparsity level of 85%.
3. **Step 3: Assign continuous interaction strengths.** For each interaction pair or cluster (since one feature in the first omic may interact with multiple features in the second omic), replace each 1 in $\mathbf{w}$ with a random value drawn from a $\mathrm{Beta}(2, 2) \times 2$ distribution.
4. **Step 4: Introduce dropout noise.**
   - Randomly zero out both vectors in an interaction pair according to $\mathbf{A}^{(12)}$.
   - Randomly zero out one of the vectors in an interaction pair according to $\mathbf{A}^{(12)}$.
   - Randomly flip non-zero elements in both $\overline{\mathbf{W}^{(1)}}$ and $\overline{\mathbf{W}^{(2)}}$ to zero, or replace zeros with random values drawn from $\mathrm{Beta}(2, 2) \times 2$.
5. **Step 5: Add Gaussian noise.** Add Gaussian noise to all non-zero elements in both $\overline{\mathbf{W}^{(1)}}$ and $\overline{\mathbf{W}^{(2)}}$.
6. **Step 6: Generate omic data matrices.** Multiply $\overline{\mathbf{W}^{(1)}}$ and $\overline{\mathbf{W}^{(2)}}$ by a latent matrix $\mathbf{H}$, generated from the $\log(1 + x)$ transformation of a negative binomial distribution, to obtain $\overline{\mathbf{X}^{(1)}}$ and $\overline{\mathbf{X}^{(2)}}$.
7. **Step 7: Apply post-processing noise.** Repeat Steps 4–6 on $\overline{\mathbf{X}^{(1)}}$ and $\overline{\mathbf{X}^{(2)}}$ to produce the final observed matrices $\mathbf{X}^{(1)}$ and $\mathbf{X}^{(2)}$.
8. **Step 8: Model inference and evaluation.** Apply the model to estimate $\widehat{\mathbf{W}}^{(1)}$ and $\widehat{\mathbf{W}}^{(2)}$, then compute the pairwise absolute cosine similarity matrix for performance evaluation.

To evaluate the models, the following metrics were used:

- **Mean and median cosine similarity:** Average, median of the absolute value of cosine similarity computed separately for interacting and non-interacting feature pairs, as well as the difference between them.
- **Mann-Whitney U test:** Statistical comparison of cosine similarity distributions between interacting and non-interacting pairs, reported as the U-test p-value.
- **DeLong test:** Evaluation based on the receiver operating characteristic (ROC) analysis, including:
  - **DeLong AUC with covariance** — a higher AUC indicates better separation between interacting and non-interacting pairs.
  - **DeLong p-value** — tests the null hypothesis H_0: AUC $= 0.5$.

The simulated data statistics and similarity/correlation distribution can be found on Figure S12 are presented in Table S5 and Figure S13.

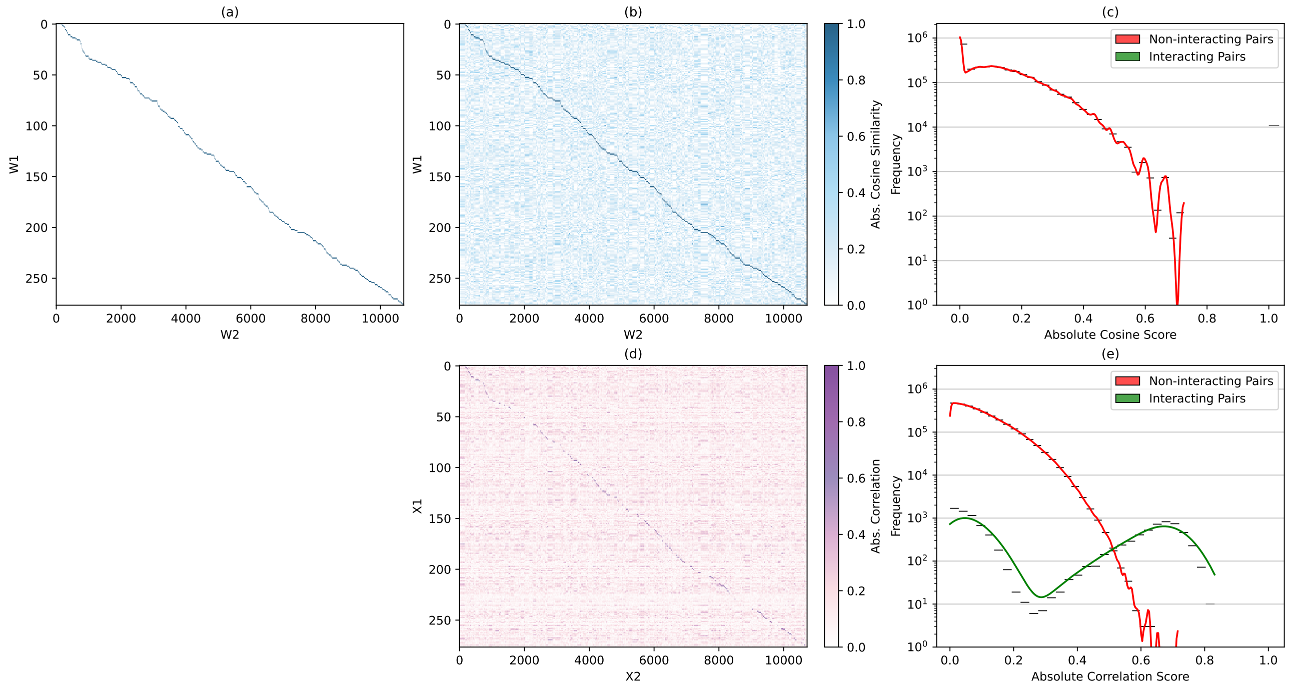

Figure S12: Original simulated data statistics and similarity/correlation distributions. (a) Heatmap of the interaction matrix $\mathbf{A}^{(12)}$. (b) Heatmap of pairwise absolute cosine similarity for interacting and non-interacting feature pairs in the original structures $\overline{\mathbf{W}}^{(1)}$ and $\overline{\mathbf{W}}^{(2)}$. (c) Distribution of cosine similarity between interacting and non-interacting feature pairs based on $\overline{\mathbf{W}}^{(1)}$ and $\overline{\mathbf{W}}^{(2)}$. (d) Heatmap of pairwise Pearson correlation between the generated data $\mathbf{X}^{(1)}$ and $\mathbf{X}^{(2)}$ after noise injection. (e) Distribution of Pearson correlation between interacting and non-interacting feature pairs derived from the noisy generated data $\mathbf{X}^{(1)}$ and $\mathbf{X}^{(2)}$.

**Metrics**	iGMFNA	iNMF	MOFA2	X-IntNMF
Median Cosine (Interacting)	0.500	0.494	0.258	0.999
Median Cosine (Non-Interacting)	0.339	0.336	0.080	0.131
Median Cosine Difference	0.161	0.158	0.178	0.868
Mean Cosine (Interacting)	0.594	0.600	0.422	0.998
Mean Cosine (Non-Interacting)	0.345	0.342	0.009	0.163
Mean Cosine Difference	0.249	0.258	0.413	0.835
Mann-Whitney p-value	0	0	0	0
DeLong AUC	0.753	0.764	0.725	0.9995
DeLong Variance	9×10^{-6}	8×10^{-6}	1×10^{-5}	4×10^{-12}
AUC p-value (vs. 0.5)	0	0	0	0

Table S5: Detailed simulation result against baselines.

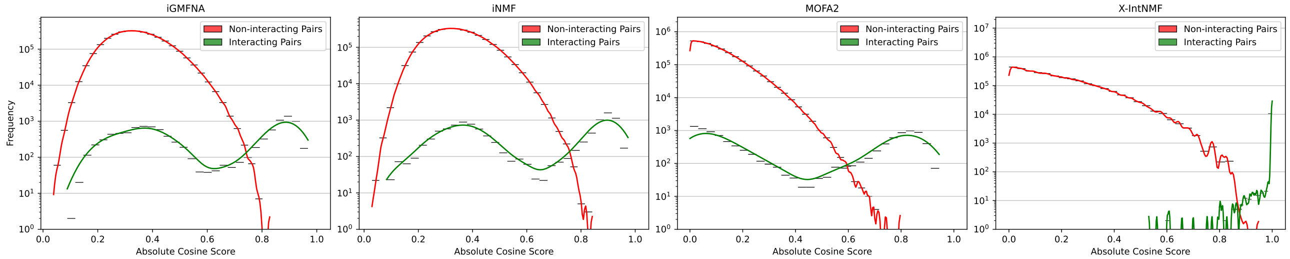

Figure S13: Distribution of the absolute cosine similarity between interacting and non-interacting feature pairs based on the predicted factor matrices $\widehat{\mathbf{W}}^{(1)}$ and $\widehat{\mathbf{W}}^{(2)}$ from different models: (a) iGMFNA; (b) iNMF; (c) MOFA; (d) X-IntNMF.

7 Survival and disease-free analysis on TCGA datasets

To further demonstrate the capability of the proposed X-IntNMF model in capturing clinically relevant patterns, we conducted comparative survival and disease-free analyses across multiple TCGA cancer datasets, benchmarking against MOFA, iNMF, and iGMFNA. For this evaluation, we followed the dataset recommendations from [1] for both overall survival and disease-free settings, as summarized in Table S6. All datasets were retrieved from the UCSC Xena Hub, and preprocessing steps were performed as described in the Data Preparation section of the main text.

For each dataset, 100 test cases were generated by randomly splitting the data into 80% training and 20% testing subsets. Within each test case, survival and disease-free analyses were performed following the procedures outlined previously, comparing X-IntNMF with the three baseline models. Model performance was evaluated based on the number of test cases yielding log-rank p-values below 0.01.

The results, summarized in Tables S7 and S8 show that X-IntNMF consistently outperforms the baselines, achieving the highest number of cancer types with statistically significant survival and disease-free associations.

Dataset	2-omics setting					3-omics setting					
	Sample size			Feature size		Sample size			Feature size		
	Raw	OS	DFI	mRNA	miRNA	Raw	OS	DFI	mRNA	miRNA	DNAm
ACC	78	78	45	10466	274	78	78	45	10466	274	12000
BLCA	423	422	192	10481	277	421	420	191	10481	277	12000
BRCA	830	829	706	10480	277	674	673	565	10480	277	12000
CESC	306	306	176	10478	277	306	306	176	10478	277	12000
CHOL	45	45	32	10449	270	45	45	32	10449	270	12000
COAD	261	259	91	10477	276	257	255	91	10477	276	12000
ESCA	192	192	91	10481	277	190	190	90	10481	277	12000
HNSC	521	520	123	10481	278	497	496	119	10481	278	12000
KICH	89	89	42	10471	275	65	65	29	10463	268	12000
KIRC	311	311	83	10479	275	201	201	68	10475	275	12000
KIRP	318	317	187	10479	275	294	293	176	10479	274	12000
LGG	524	522	134	10480	278	524	522	134	10480	278	12000
LIHC	415	414	351	10479	278	406	405	343	10479	278	12000
LUAD	466	457	282	10481	277	456	447	273	10481	277	12000
LUSC	373	332	226	10481	278	338	331	226	10481	278	12000
MESO	87	86	15	10469	277	87	86	15	10469	277	12000
OV	304	303	146	10479	274	295	294	139	10479	270	12000
PAAD	182	182	72	10480	277	182	182	72	10480	277	12000
PRAD	543	543	379	10480	277	526	526	364	10480	277	12000
READ	92	91	29	10469	274	88	87	28	10469	274	12000
SARC	258	258	153	10480	277	258	258	153	10480	277	12000
STAD	403	398	255	10481	278	367	363	236	10481	278	12000
THCA	567	567	398	10479	278	558	558	390	10479	278	12000
UCEC	194	183	123	10479	278	193	182	122	10479	278	12000
UCS	56	56	27	10475	277	56	56	27	10475	277	12000

Table S6: Statistics of TCGA datasets used in survival and disease-free analyses. The counts of mRNA, miRNA, and DNA methylation features are denoted as mRNA, miRNA, and DNAm, respectively. The sample sizes for overall survival (OS) and disease-free interval (DFI) analyses are also provided.

Dataset	2-omics setting				3-omics setting			
	X-IntNMF	MOFA2	iGMFNA	iNMF	X-IntNMF	MOFA2	iGMFNA	iNMF
Total (25)	**18**	3	1	4	**15**	6	6	5
ACC	**26**	23	17	18	23	24	18	**26**
BLCA	**36**	26	23	32	18	**30**	9	10
BRCA	**18**	10	2	8	**32**	19	6	10
CESC	**14**	10	2	5	10	11	**13**	7
CHOL	1	1	**2**	**2**	0	**1**	0	0
COAD	2	**3**	2	0	**3**	0	0	2
ESCA	**5**	0	3	1	**2**	1	**2**	**2**
HNSC	**30**	28	5	14	13	6	**14**	7
KICH	0	0	0	0	**1**	**1**	0	0
KIRC	**37**	31	4	26	**14**	4	9	6
KIRP	**16**	8	9	11	10	**15**	7	5
LGG	**85**	65	83	76	90	**93**	70	75
LIHC	**21**	16	6	15	**14**	5	5	5
LUAD	**11**	5	9	8	**19**	4	15	8
LUSC	4	**5**	0	0	**2**	1	1	1
MESO	30	**41**	17	26	**37**	29	10	28
OV	**3**	0	0	1	1	1	**4**	3
PAAD	**9**	4	2	4	7	5	1	**17**
PRAD	**5**	1	0	0	**2**	0	0	0
READ	**1**	0	0	**1**	**1**	0	0	0
SARC	**19**	5	12	8	**26**	14	9	6
STAD	**11**	1	2	2	2	3	2	**10**
THCA	**2**	0	0	0	**2**	0	0	0
UCEC	5	3	1	**8**	**4**	0	**4**	1
UCS	1	2	0	**3**	**1**	**1**	**1**	**1**

Table S7: Overall survival analysis results for both 2-omics and 3-omics settings on various TCGA datasets. The datasets are chosen based on the availability of survival data and the recommendation from [1]. The best results are highlighted in blue.

**Dataset**	2-omics setting				3-omics setting			
	X-IntNMF	*MOFA2*	*iGMFNA*	*iNMF*	*X-IntNMF*	*MOFA2*	*iGMFNA*	*iNMF*
Total (23)	**16**	2	5	3	**16**	5	3	5
ACC	**1**	0	0	**1**	**2**	0	0	1
BLCA	**2**	0	0	0	**3**	0	0	0
BRCA	**1**	0	0	**1**	**2**	1	0	0
CESC	2	1	**3**	2	**2**	**2**	0	0
CHOL	0	0	0	**1**	0	0	0	0
COAD	**3**	0	0	0	**1**	0	**1**	0
ESCA	1	**3**	0	0	**1**	**1**	0	**1**
HNSC	**1**	0	0	0	**1**	0	0	0
KIRC	0	0	0	0	**1**	0	0	0
KIRP	5	4	**17**	4	3	5	2	**6**
LGG	**4**	3	0	0	**2**	0	1	0
LIHC	**11**	6	3	2	**11**	10	4	3
LUAD	**3**	1	1	0	**2**	0	0	0
LUSC	**2**	0	**2**	0	**1**	**1**	0	**1**
OV	2	1	**4**	0	1	1	**3**	0
PAAD	**7**	2	4	5	4	7	4	**11**
PRAD	**23**	7	3	11	**5**	4	3	0
SARC	**6**	3	**6**	3	4	**9**	3	1
STAD	**13**	6	0	0	**14**	11	1	2
TGCT	**1**	0	0	0	**2**	**2**	0	**2**
THCA	**8**	5	0	0	2	1	**3**	0
UCEC	**1**	**1**	0	0	**1**	0	0	0

Table S8: Disease-free analysis results for both 2-omics and 3-omics settings on various TCGA datasets. The datasets are chosen based on the availability of survival data and the recommendation from [1]. The best results are highlighted in blue.

8 Gene enrichment and KEGG pathway analysis

To verify the biological relevance of the learned feature representations $\mathbf{W}^{(d)}$ from X-IntNMF, we conducted a biological interpretability analysis on the mRNA omic layer of the breast cancer dataset. Specifically, we focused on the top 15 gene features from each latent component learned by X-IntNMF, concatenated across all omics types. The enrichment analysis was performed using the DAVID Knowledgebase [2]. The enriched KEGG pathways and Gene Ontology (GO) terms are summarized in Table S9.

Many of the enriched pathways and GO terms are known to be associated with breast cancer. Nicotine addiction, through activation of nicotinic acetylcholine receptors, has been shown to promote proliferation, epithelial–mesenchymal transition, angiogenesis, and therapeutic resistance in breast cancer cells, implicating neurotransmitter-related signaling in oncogenesis [3]. Membrane-bound ATP-binding cassette (ABC) transporters play a dual role in breast cancer by enabling multidrug efflux (chemoresistance) and by participating in oncogenic signaling networks that support tumor progression and metastasis [4]. Meanwhile, T cell receptor (TCR) signaling regulates antitumor immune responses, and in breast cancer, the diversity and clonality of TCR repertoires correlate with prognosis, highlighting the importance of immune–microenvironment interactions [5]. Synaptic-related processes such as GABAergic synapse signaling and the synaptic vesicle cycle may also be co-opted in the breast tumor microenvironment. For instance, GABA receptors and transporters are upregulated in breast cancer brain metastases, suggesting that somatodendritic compartment signaling and monoatomic ion channel activity (e.g., chloride or $\mathrm{GABA_A}$/$\mathrm{GABA_B}$ receptor channels) modulate tumor–nerve interactions and enhance metastasis [6].

Category	Term	Pvalue	FDR
GO Biological Process	multicellular organismal process	8.400E-12	3.190E-08
	multicellular organism development	1.020E-10	1.590E-07
	cell-cell signaling	1.260E-10	1.590E-07
	system development	1.370E-09	1.290E-06
	chemical synaptic transmission	3.910E-09	2.470E-06
GO Cellular Component	somatodendritic compartment	4.410E-08	1.890E-05
	neuron projection	3.400E-07	7.290E-05
	monoatomic ion channel complex	7.420E-07	1.060E-04
	synaptic membrane	1.080E-06	1.160E-04
	plasma membrane region	1.410E-06	1.210E-04
GO Molecular Function	monoatomic ion channel activity	7.740E-08	4.530E-05
	passive transmembrane transporter activity	1.780E-07	4.530E-05
	channel activity	1.780E-07	4.530E-05
	transmembrane transporter activity	5.660E-07	1.080E-04
	gated channel activity	8.860E-07	1.350E-04
KEGG Pathway	Nicotine addiction	1.680E-04	2.890E-02
	ABC transporters	2.630E-04	2.890E-02
	T cell receptor signaling pathway	9.680E-04	7.100E-02
	GABAergic synapse	5.720E-03	3.140E-01
	Synaptic vesicle cycle	1.870E-02	7.260E-01

Table S9: Gene set enrichment analysis for the top genes identified by X-IntNMF on the BRCA dataset. The table lists significantly enriched Gene Ontology (GO) terms and KEGG pathways along with their associated p-values and false discovery rates (FDR).

References

[1] J. Liu, T. Lichtenberg, K. A. Hoadley, L. M. Poisson, A. J. Lazar, A. D. Cherniack, A. J. Kovatich, C. C. Benz, D. A. Levine, A. V. Lee, et al. "An integrated TCGA pan-cancer clinical data resource to drive high-quality survival outcome analytics". In: *Cell* 173.2 (2018), pp. 400–416.

[2] B. T. Sherman, M. Hao, J. Qiu, X. Jiao, M. W. Baseler, H. C. Lane, T. Imamichi, and W. Chang. "DAVID: a web server for functional enrichment analysis and functional annotation of gene lists (2021 update)". In: *Nucleic acids research* 50.W1 (2022), W216–W221.

[3] Z. Khodabandeh, M. Valilo, K. Velaei, and A. Pirpour Tazehkand. "The potential role of nicotine in breast cancer initiation, development, angiogenesis, invasion, metastasis, and resistance to therapy". In: *Breast Cancer* 29.5 (2022), pp. 778–789.

[4] L. Duvivier, L. Gerard, A. Diaz, and J.-P. Gillet. "Linking ABC transporters to the hallmarks of cancer". In: *Trends in Cancer* 10.2 (2024), pp. 124–134.

[5] T. Wang, C. Wang, J. Wu, C. He, W. Zhang, J. Liu, R. Zhang, Y. Lv, Y. Li, X. Zeng, et al. "The different T-cell receptor repertoires in breast cancer tumors, draining lymph nodes, and adjacent tissues". In: *Cancer immunology research* 5.2 (2017), pp. 148–156.

[6] Q. Cui, D. Jiang, Y. Zhang, and C. Chen. "The tumor-nerve circuit in breast cancer". In: *Cancer and Metastasis Reviews* 42 (2023), pp. 543–574. DOI: 10.1007/s10555-023-10095-1.
